# A multi-country study on the impact of sex and age on oral features of COVID-19 infection in adolescents and young adults

**DOI:** 10.1186/s12903-022-02515-5

**Published:** 2022-11-19

**Authors:** Heba Jafar Sabbagh, Wafaa Abdelaziz, Maryam Quritum, Rana Abdullah Alamoudi, Nada Abu Bakr AlKhateeb, Joud Abourdan, Nafeesa Qureshi, Shabnum Qureshi, Ahmed H. N. Hamoud, Nada Mahmoud, Ruba Odeh, Nuraldeen Maher Al-Khanati, Rawiah Jaber, Abdulrahman Loaie Balkhoyor, Mohammed Shabi, Morenike Oluwatoyin Folayan, Omolola Alade, Noha Gomaa, Raqiya Alnahdi, Nawal A. Mahmoud, Hanane El Wazziki, Manal Alnaas, Bahia Samodien, Rawa A. Mahmoud, Nour Abu Assab, Sherin Saad, Maha Mohamed Alsayad, Sondos G. Alhachim, Maha El Tantawi

**Affiliations:** 1 Pediatric Dentistry Department, Faculty of Dentistry, King Abdulaziz University, P.O. Box: 80200, Jeddah, 21589 Saudi Arabia; 2 Department of Pediatric Dentistry and Dental Public Health, Faculty of Dentistry, Alexandria University, Alexandria, 21561 Egypt; 3 Pediatric Dentistry Department, Faculty of Dentistry, King Abdulaziz University, Jeddah, 21589 Saudi Arabia; 4 Faculty of Medicine, King Abdulaziz University, Jeddah, 21589 Saudi Arabia; 5 Medical Faculty, Istanbul Medipol University, 34230 Istanbul, Turkey; 6 City Quay Dental Practice and Implant Centre, Dundee, DD1 3JA UK; 7 Department of Education, University of Kashmir, Srinagar, 190006 India; 8 Faculty of Dentistry, Alexandria University, Alexandria, 21561 Egypt; 9 Faculty of Dentistry, National Ribat University, 1111 Khartoum, Sudan; 10 College of Dentistry, Ajman University, Ajman, United Arab Emirates; 11 Department of Oral and Maxillofacial Surgery, Faculty of Dentistry, Syrian Private University, Damascus, 386 Syria; 12 General Courses, King Abdulaziz University, Jeddah, 21589 Saudi Arabia; 13 Faculty of Engineering, King Abdulaziz University, Jeddah, 21589 Saudi Arabia; 14 University of Jeddah, Jeddah, 23218 Saudi Arabia; 15 Department of Child Dental Health, Obafemi Awolowo University, Ile-Ife, Osun State Nigeria; 16 Department of Preventive and Community Dentistry, Faculty of Dentistry, Obafemi Awolowo University, Ile-Ife, Nigeria; 17 Schulich School of Medicine and Dentistry, University of Western Ontario, London, Ontario Canada; 18 Department of Dental Surgery, Oman Dental College, 116 Muscat, Oman; 19 Institute of Creative Art and Design (ICAD), Kuala Lumpur Campus, UCSI University, 56000 Kuala Lumpur, Malaysia; 20 Department of Cereal Plant Pathology, National Institute of Agricultural Research, 10090 Rabat, Morocco; 21 Division of Imaging Science and Technology, School of Medicine, University of Dundee, Dundee, DD1 4HN UK; 22 Western Cape Education Department, Cape Town, 8001 South Africa; 23 Musculoskeletal Center, International Medical Centre, Jeddah, 21451 Saudi Arabia; 24 Schools of Awqaf, Directorate of Education, Jerusalem, Israel; 25 Department of Pharmacology, Institute of Neuroscience and Physiology, University of Gothenburg, Box 431, 40530 Gothenburg, Sweden; 26 Center of Orthopedics and Spine Technologies, Jeddah, Saudi Arabia; 27 Health Education Services, Ingham County, Lansing, MI 48933 USA

**Keywords:** COVID-19 infection, Multi-country, Oral conditions, Adolescents

## Abstract

**Background:**

Oral diseases are features of COVID-19 infection. There is, however, little known about oral diseases associated with COVID-19 in adolescents and young adults (AYA). Therefore, the aim of this study was to assess oral lesions’ association with COVID-19 infection in AYA; and to identify if sex and age will modify these associations.

**Methodology:**

Data was collected for this cross-sectional study between August 2020 and January 2021 from 11-to-23 years old participants in 43-countries using an electronic validated questionnaire developed in five languages. Data collected included information on the dependent variables (the presence of oral conditions- gingival inflammation, dry mouth, change in taste and oral ulcers), independent variable (COVID-19 infection) and confounders (age, sex, history of medical problems and parents’ educational level). Multilevel binary logistic regression was used for analysis.

**Results:**

Complete data were available for 7164 AYA, with 7.5% reporting a history of COVID-19 infection. A significantly higher percentage of participants with a history of COVID-19 infection than those without COVID-19 infection reported having dry mouth (10.6% vs 7.3%, AOR = 1.31) and taste changes (11.1% vs 2.7%, AOR = 4.11). There was a significant effect modification in the association between COVID-19 infection and the presence of dry mouth and change in taste by age and sex (*P* = 0.02 and < 0.001).

**Conclusion:**

COVID-19 infection was associated with dry mouth and change in taste among AYA and the strength of this association differed by age and sex. These oral conditions may help serve as an index for suspicion of COVID-19 infection in AYA.

**Supplementary Information:**

The online version contains supplementary material available at 10.1186/s12903-022-02515-5.

## Introduction

Oral lesions are associated with several systemic or local infections caused by micro-organisms. These include primary and secondary viral pathological lesions associated with oral infective diseases and dysplastic-neoplastic transformations [1]. These oral lesions cause distress, physical and psychological disabilities, as well as functional limitation [2].

COVID-19 infection is associated with oral lesions such as gustatory impairment either due to the effect of the virus on oral tissues or as secondary manifestations resulting from it [3]. Other identified oral lesions include Aphthous-like ulcer, erythema, and lichen planus [4].

The oral lesions associated with COVID-19 infection have been reported in adults. There are, however, differences in the clinical manifestations of COVID-19 in children compared to adults [5, 6] which may imply differences in the presence of oral lesions associated with COVID-19 infection by age. However, there is little known about the oral features of COVID-19 among adolescents and young adults (AYA). Also, there is limited known about sex differences in the oral features of COVID-19 infection among AYA. Yet, identifying sex and age differences in the oral features of COVID-19 is important for characterising the lesions, and enabling health care providers to improve the oral health management for AYA. Therefore, the aim of the present study was to assess the associations between some oral conditions and COVID-19 infection in AYA; and to investigate sex and age differences in these associations. The null hypothesis of the study was that no oral conditions would be associated with COVID-19 infection; and age and sex would not modify this association.

## Methods

### Study design

This was a secondary analysis of data from cross-sectional study conducted between August 2020 to January 2021. The aim of the primary study was to assess the change in use of regular and electronic cigarettes during the COVID-19 pandemic among AYA [7].

### Participants and Setting

Data was collected from 11 to 23-year old participants recruited through electronic data collection from various countries. Participants were included if they/ their parents consented to participate, if they could read and understand the study tool and if they could access the survey on an electronic device using internet access.. Ethical approval was sought from the Research Ethics Committee of the Faculty of Dentistry, King Abdulaziz University in Saudi Arabia (#13–08-20) and Nigeria (IPH/OAU/12/1604). The study was carried out according to the Helsinki Declaration [8]. Informed consent was obtained from parents of participants who reported they were aged 11 to 17 years old; and assent was also sought from the participants before they could continue with the study participation. Respondents aged 18 to 23 years could participate by giving their own informed consent.

### Sample size

Sample size of the original study was determined using the frequency of using regular and electronic cigarettes. This was estimated to range from 4.8 to 36.6% [9]. The sample size needed for the primary study was 356. For the present study, we also made sure that the data of at least 500 participants were available for the regression analysis [10].

### Data sources/ measurement

The administered questionnaire began by explaining the purpose of the study, assuring participant of the confidentiality of their responses, and the freedom to withdraw from the survey at any time. The questionnaire consisted of three sections: The first section collected data on the participant including age (categorized into 11–14, 15–17 and 18–23 years old), sex at birth (male and female) and educational level (elementary school or none, middle, high school or university) as well as on the mother’s and father’s educational level (also elementary school or none, middle, high school or university).

The second section assessed whether the participant had a history of medical problems (yes/ no) and a history of COVID-19 infection (yes/ no). These questions were adapted from previous research [11].

The third section had participants check a list of possible oral lesions they may have had during the pandemic. A tick of the checkbox indicated that they had the oral lesion(s). Study participants could check multiple responses. These lesions/conditions included gingival inflammation, dry mouth, change in taste, and oral ulcers. These conditions were based on those previously reported to be associated with COVID-19 in adults or older adults [3].

The questionnaire was developed in Arabic and translated by native speakers into French, Malay, Turkish and English. The content validity index (CVI) was calculated for the translated versions [12]. For the English and Arabic versions, nine dentists evaluated the questionnaire. The CVI for both versions was 0.87. The Turkish version was evaluated by seven dentists and the CVI was 0.97. The Malay and French versions were evaluated by five dentists each and the CVI was 0.80 and 0.88, respectively. Each version was pilot tested by 10 participants for clarity and cultural suitability of terms. The questionnaire was finally uploaded to SurveyMonkey® online platform, and a tailored link was made for each collaborator in each country. The snowballing technique was used to recruit participants: Twenty-five collaborators circulated the link to the questionnaire in their networks and requested the participants to circulate the questionnaire further within their networks.

### Statistical analysis

Descriptive statistics as frequencies and percentages were calculated for the study variables. The associations between the presence of oral conditions and COVID-19 infection were assessed using the Chi-square test. Multilevel binary logistic regression modelling using IBM SPSS for Windows version 22.0 (IBM Corp., Armonk, N.Y., USA) was used to assess the associations between the presence of oral conditions including gingival inflammation, dry mouth, change in taste and oral ulcers (dependent variable) and COVID-19 infection (independent variable) controlling for confounders (parental education, having medical problem, participants’ sex and age groups) which were all introduced as fixed effect variables whereas countries were introduced as random effect variables to account for the variability among countries in the dependent variables caused by various factors such as country income level, dentist availability, healthcare systems characteristics and others. The interaction between COVID-19 infection and age and sex respectively was also assessed by calculating the *p* value for the interaction between these terms (age and COVID-19 infection and sex and COVID-19 infection) in the regression models. Adjusted odds ratio (AORs), 95% confidence interval (CIs) and *p*-values were calculated. Statistical significance was set at p-value less than 0.05.

## Results

Complete data were available for 7164 participants from 43 countries. Table 1 shows that 4109 (57.4%) participants were females, 5342 (74.6%) were 18–23 years old, 4079 (56.9%) were university educated and more than 40% of participants had mothers or fathers with university or higher education. Also, 925 (12.9%) reported having medical problems and 540 (7.5%) were infected with COVID-19.

**Table 1:** Socio-demographic and COVID-19 status in AYA from 43 countries (*n* = 7164)

Factors		N (%)
Sex	Male	3055 (42.6)
	Female	4109 (57.4)
Age	11–14 years	709 (9.9)
	15–17 years	1113 (15.5)
	18–23 years	5342 (74.6)
Participant’s education	Elementary	238 (3.3)
	Middle	588 (8.2)
	High school	2259 (31.5)
	University	4079 (56.9)
Mother’s education	None	576 (8.0)
	Elementary/ middle	1522 (21.2)
	High school	2022 (28.2)
	University or higher	3044 (42.5)
Father’s education	None	325 (4.5)
	Elementary/ middle	1244 (17.4)
	High school	1786 (24.9)
	University or higher	3809 (53.2)
Has medical problems	Yes	925 (12.9)
	No	6239 (87.1)
Infected with COVID-19	Yes	540 (7.5)
	No	6624 (92.5)

Table 2 shows that 4140 (57.8%) participants reported no oral conditions. The most frequently reported oral conditions were gingival inflammation (10.7%) and dry mouth (7.5%). Change in taste was reported by 238 (3.3%) participants.

**Table 2:** Association between oral conditions and COVID-19 infection status among AYA (*n* = 7164)

	Total	Infected with COVID-19 *N* = 540	Not infected with COVID-19 *N* = 6624	*P* value
Nothing	4140 (57.8)	259 (48.0)	3881 (58.6)	< 0.001
Gingival inflammation	767 (10.7)	71 (13.1)	696 (10.5)	0.06
Dry mouth	539 (7.5)	57 (10.6)	482 (7.3)	0.008
Change in taste	238 (3.3)	60 (11.1)	178 (2.7)	< 0.001
Burns or ulcers	89 (1.2)	13 (2.4)	76 (1.1)	0.01

A significantly higher percentage of participants with COVID-19 than those who had no COVID-19 infection reported having dry mouth (10.6% vs 7.3%, *p* = 0.008), change in taste (11.1% vs 2.7%, *p* < 0.001) and oral ulcers (2.4% vs 1.1%, *p* = 0.01). A significantly higher percentage of participants with no history of COVID-19 infection than those with an infection reported having no oral conditions during the pandemic (58.6% vs 48.0%, *p* < 0.001).

Table 3 shows the results of the multilevel binary logistic regression analysis for the association between the presence of oral conditions and COVID-19 infection reported by participants. COVID-19 infection was associated with significantly higher odds of the presence of dry mouth (AOR = 1.31, *p* = 0.002) and change in taste (AOR = 4.11, *p* < 0.001).

**Table 3:** Differences by sex and age groups in the association between COVID-19 infection and reported oral conditions using multilevel analysis

Dependent variables	Association with COVID-19 infection		Association with COVID-19 infection by age			P for interaction with age	Association with COVID-19 infection by sex		P for interaction with sex
	AOR (95%CI)	*P* value	18–23	15–17	11–14		Male	Female	
Gingival inflammation	1.17 (0.89, 1.53)	0.26	1.11 (0.76, 1.62)	1.94 (1.19, 3.15)*	1.73 (0.82, 3.66)	0.63	1.30 (0.85, 1.98)	1.09 (0.82, 1.45)	0.04*
Dry mouth	1.31 (1.11, 1.55)	0.002*	1.31 (1.10, 1.56)*	1.65 (0.80, 3.39)	1.48 (0.42, 5.20)	0.02*	1.42 (1.07, 1.89)*	1.25 (0.94, 1.66)	0.02*
Change in taste	4.11 (3.28, 5.16)	< 0.001*	2.70 (2.19, 3.34)*	5.79 (2.76, 12.16)*	2.28 (0.97, 5.35)	< 0.001*	2.46 (2.10, 2.89)*	4.92 (3.07, 7.96)*	< 0.001*
Burns or ulcers	1.20 (0.81, 1.79)	0.37	1.17 (0.80, 1.70)	1.24 (0.80, 1.92)	1.70 (0.63, 4.59)	0.96	1.22 (0.75, 2.00)	1.18 (0.83, 1.69)	0.61

Adjusted for medical problems, and parents’ education

*: statistically significant at *P* < 0.05

Age significantly modified the association between COVID-19 infection and the presence of dry mouth: AYA 18 to 23-year-olds had significantly lower odds of having dry mouth associated with COVID-19 infection than 15 to17-year-olds and 11 to14-years-olds (AOR = 1.31 vs 1.64 and 1.48, *p* = 0.02). Age also significantly modified the association between change in taste and COVID-19 infection: 15–17-year-olds had higher odds of having a change in taste associated with COVID-19 infection than 18–23-year-olds and 11–14-year-olds (AOR = 5.79 vs 2.70 and 2.28; *p* < 0.001).

Sex significantly modified the association between the presence of gingival inflammation and COVID-19 infection: males had significantly higher odds of the association than females (AOR = 1.30 vs 1.09; *p* = 0.04). Sex also modified the association between dry mouth and COVID-19 infection: males had higher odds of having an association than females (AOR = 1.42 vs 1.25; p = 0.02). In addition, sex modified the association between change in taste and COVID-19 infection: females had higher odds of this association than males (AOR = 4.92 vs 2.46; *p* < 0.001).

## Discussion

The findings showed that about 4 in 10 AYA reported oral conditions during the pandemic with change in taste, dry mouth and oral ulcers positively associated with COVID-19 infection. The associations of COVID-19 infection with dry mouth and change in taste were modified by age: there was a stronger association between COVID-19 infection, dry mouth and change in taste among 15–17-year-old participants than among other age groups. Sex also modified the associations of COVID-19 infection with dry mouth and change in taste: males had higher odds for having dry mouth than females, while females had higher odds of having a change in taste than males. The null hypothesis of the study was, therefore, not supported.

One of the strengths of the study was that we provided information on the possible oral features of COVID-19 infection in AYA aged 11 to 23 years; and identified how sex and age modified these associations. Also, the study recruited a large sample of participants from countries representing various income-levels and regions thereby increasing the generalizability and applicability of findings to various settings. The study, however, has some limitations. The oral conditions were self-reported thereby introducing subjectivity to the reporting with the risk of under-estimation of lesions like gingival inflammation and dry mouth. Although a clinical examination might be considered more objective, it was not possible to conduct clinical assessment of study participants because of the restrictions with contacts during the pandemic. This was the case in most of studies assessing oral lesions during the COVID-19 pandemic [3]. However, a study conducted to assess self-report for disease surveillance reported a satisfactory outcomes for patent’s’ self-reporting their disease category [13]. We, additionally, focused on oral conditions that patients are likely able to self-report thereby reducing assessment bias. The data was based on a convenience sample driven by snowball sampling where participants refer the survey link to people they know because data collection was restricted to online methods during the pandemic. These non-probability samples introduce some bias as only participants who have a smartphone and who can access the internet could participate in the study. Such participants are not similar to the general population in each country which may affect the generalizability of findings. In addition, the study is cross sectional and cannot, therefore, prove causality.

The association between COVID-19 infection and change in taste and dry mouth had been reported and explained in previous studies [14–16]. However, none of them discussed these changes in AYA. Taste disorders, oral ulcerations, desquamative gingivitis, candidiasis [17–19], halitosis, erythema, and spontaneous bleeding from the gingiva [19] were also associated with COVID-19 infection. In a symptomatic paediatric cohort, taste alterations were also reported [20]. These oral conditions may be the side effects of SARS-CoV-2 infection, or may be the sequel to super-infections, immuno-compromise, or complications of medications used to manage COVID-19 infection [21]. Psychological stress, a complication of the COVID-19 pandemic [22, 23], and poor oral hygiene during the pandemic might also play a part in the manifestation of gingival inflammation, dry mouth, and ulcers. Some lesions may be attributed to ACE2 receptor of SARS-CoV-2 found in the mucosa acting as a gateway to COVID-19 infection [24].

The association between COVID-19 infection and gingival inflammation and dry mouth was stronger in males than females in the present study. While a male predilection was noticed in the current study, previous research showed that these symptoms were more frequent in females than males [5, 25–27]. It is important to note that these studies were all conducted on adults. This emphasizes the importance of the present study finding suggesting gender differences in younger age groups. Further studies including AYA are needed to confirm this relationship. On the other hand, females showed a stronger association between COVID-19 infection and taste changes like a prior study by Galmiche, et al. [28] which reported a higher alteration in taste among females compared to males. Taste perception can vary by sex, probably because females have more fungiform papillae and taste buds than males [29].

In the present study, COVID-19 infection had the strongest association with altered taste in 15–17-year-olds and the oldest age group of 18–23-year-old showed the weakest association between COVID-19 infection and reporting dry mouth. Direct comparison with previous studies is difficult because these studies were either case-reports or were conducted in adults [27, 30]. However, older children were reported to be more able to identify smells and tastes than younger children [31] and thus, more able to detect loss of taste than younger children. Further research is needed to explain the age-related differences in AYA groups.

## Conclusion

Participants who had COVID-19 infection were more likely to report change in taste, dry mouth and oral ulcers. Sex and age moderated the associations between COVID-19 infection, dry mouth and change in taste in AYA. These findings may be useful for screening for COVID-19 infection among AYA.

## Supplementary Information

**Additional file 1.**

**Additional file 2.**

## Data Availability

The datasets used and/or analysed during the current study are available from the corresponding author on reasonable request.

## Abbreviations

AYA: Adolescents and young adults
CVI: Content validity index
AOR: Adjusted odds ratio
CI: Confidence interval

## References

[1] Lynch DP, Tyring S (2006). Oral manifestations of viral dieases. Mucosal Immunology and Virology.

[2] Villanueva-Vilchis MD, López-Ríos P, García IM, Gaitán-Cepeda LA (2016). Impact of oral mucosa lesions on the quality of life related to oral health. An etiopathogenic study. Med Oral Patol Oral Cir Bucal.

[3] Amorim Dos Santos J, Normando AGC, Carvalho da Silva RL, Acevedo AC, De Luca CG, Sugaya N (2021). Oral Manifestations in Patients with COVID-19: A Living Systematic Review. J Dent Res.

[4] Fidan V, Koyuncu H, Akin O (2021). Oral lesions in Covid 19 positive patients. Am J Otolaryngol.

[5] Chen A, Huang JX, Liao Y, Liu Z, Chen D, Yang C (2020). Differences in Clinical and Imaging Presentation of Pediatric Patients with COVID-19 in Comparison with Adults. Radiol Cardiothorac Imaging.

[6] Girona-Alarcon M, Bobillo-Perez S, Sole-Ribalta A, Hernandez L, Guitart C, Suarez R (2021). The different manifestations of COVID-19 in adults and children: a cohort study in an intensive care unit. BMC Infect Dis.

[7] Sabbagh HJ, Abdelaziz W, Quritum M, AlKhateeb NA, Abourdan J, Qureshi N (2022). Cigarettes' use and capabilities-opportunities-motivation-for-behavior model: a multi-country survey of adolescents and young adults. Front Public Health.

[8] WMA. World Medical Association Declaration of Helsinki (2001). Ethical principles for medical research involving human subjects. Bull World Health Organ.

[9] World Health Organization (2019). WHO global report on trends in prevalence of tobacco use 2000–2025.

[10] Nemes S, Jonasson JM, Genell A, Steineck G (2009). Bias in odds ratios by logistic regression modelling and sample size. BMC Med Res Methodol.

[11] Folayan MO, Ibigbami O, Brown B, et al. Differences in COVID-19 Preventive Behavior and Food Insecurity by HIV Status in Nigeria. AIDS Behav. 2022;26(3):739-51. 10.1007/s10461-021-03433-3. 10.1007/s10461-021-03433-3 PMC8360820 34387776

[12] Yusoff M (2019). ABC of Content Validation and Content Validity Index Calculation. Educ Med J.

[13] Bourgeois FT, Porter SC, Valim C, Jackson T, Cook EF, Mandl KD (2007). The value of patient self-report for disease surveillance. J Am Med Inform Assoc.

[14] Mastrangelo A, Bonato M, Cinque P (2021). Smell and taste disorders in COVID-19: From pathogenesis to clinical features and outcomes. Neurosci Lett.

[15] Meunier N, Briand L, Jacquin-Piques A, Brondel L, Pénicaud L (2020). COVID 19-Induced Smell and Taste Impairments: Putative Impact on Physiology. Front Physiol.

[16] Tsuchiya H (2021). Oral Symptoms Associated with COVID-19 and Their Pathogenic Mechanisms: A Literature Review. Dent J (Basel).

[17] Amorim Dos Santos J, Normando AGC, Carvalho da Silva RL, De Paula RM, Cembranel AC, Santos-Silva AR (2020). Oral mucosal lesions in a COVID-19 patient: New signs or secondary manifestations?. Int J Infect Dis.

[18] Cebeci Kahraman F, Çaşkurlu H (2020). Mucosal involvement in a COVID-19-positive patient: A case report. Dermatol Ther.

[19] Iranmanesh B, Khalili M, Amiri R, Zartab H, Aflatoonian M (2021). Oral manifestations of COVID-19 disease: A review article. Dermatol Ther.

[20] Bardellini E, Bondioni MP, Amadori F, Veneri F, Lougaris V, Meini A (2021). Non-specific oral and cutaneous manifestations of Coronavirus Disease 2019 in children. Med Oral Patol Oral Cir Bucal.

[21] Cao B, Wang Y, Wen D, Liu W, Wang J, Fan G (2020). A Trial of Lopinavir-Ritonavir in Adults Hospitalized with Severe Covid-19. N Engl J Med.

[22] Sabbagh HJ, Abdelaziz W, Alghamdi W, Quritum M, AlKhateeb NA, Abourdan J (2022). Anxiety among Adolescents and Young Adults during COVID-19 Pandemic: A Multi-Country Survey. Int J Environ Res Public Health.

[23] Sinanović O, Muftić M, Sinanović S (2020). COVID-19 Pandemia: Neuropsychiatric Comorbidity and Consequences. Psychiatr Danub.

[24] Zhang T, Liu D, Tian D, Xia L (2021). The roles of nausea and vomiting in COVID-19: did we miss something?. J Microbiol Immunol Infect.

[25] El Tantawi M, Sabbagh HJ, Alkhateeb NA, Quritum M, Abourdan J, Qureshi N (2022). Oral manifestations in young adults infected with COVID-19 and impact of smoking: a multi-country cross-sectional study. PeerJ..

[26] Lechien JR, Chiesa-Estomba CM, De Siati DR, Horoi M, Le Bon SD, Rodriguez A (2020). Olfactory and gustatory dysfunctions as a clinical presentation of mild-to-moderate forms of the coronavirus disease (COVID-19): a multicenter European study. Eur Arch Otorhinolaryngol.

[27] Mercante G, Ferreli F, De Virgilio A, Gaino F, Di Bari M, Colombo G (2020). Prevalence of Taste and Smell Dysfunction in Coronavirus Disease 2019. JAMA Otolaryngol Head Neck Surg.

[28] Galmiche S, Bruel T, Madec Y, Tondeur L, Grzelak L, Staropoli I (2021). Characteristics Associated with Olfactory and Taste Disorders in COVID-19. Neuroepidemiology..

[29] Bartoshuk LM, Duffy VB, Miller IJ (1994). PTC/PROP tasting: anatomy, psychophysics, and sex effects. Physiol Behav.

[30] Mak PQ, Chung KS, Wong JS, Shek CC, Kwan MY (2020). Anosmia and Ageusia: Not an Uncommon Presentation of COVID-19 Infection in Children and Adolescents. Pediatr Infect Dis J.

[31] Concheiro-Guisan A, Fiel-Ozores A, Novoa-Carballal R, González-Duran ML, Portugués de la Red M, Martínez-Reglero C (2021). Subtle olfactory dysfunction after SARS-CoV-2 virus infection in children. Int J Pediatr Otorhinolaryngol.

