# Supplementary material for: A multi-country study on the impact of sex and age on oral features of COVID-19 infection in adolescents and young adults
Source: BMC Oral Health. 2022 Nov 19;22:513. doi: 10.1186/s12903-022-02515-5 (PMC9675238; doi:10.1186/s12903-022-02515-5)
Supplement: Supplementary file 1 — Additional file 1. [file 12903_2022_2515_MOESM1_ESM.docx]

**Additional File 1:** Participants by country

| **Country** | **Frequency** | **Percent** |
| --- | --- | --- |
| 1. Afghanistan | 3 | .0 |
| 1. Algeria | 7 | .1 |
| 1. Argentina | 3 | .0 |
| 1. Australia | 5 | .1 |
| 1. Bahrain | 15 | .2 |
| 1. Belgium | 7 | .1 |
| 1. Canada | 206 | 2.9 |
| 1. Chad | 12 | .2 |
| 1. China | 4 | .1 |
| 1. Cyprus | 11 | .2 |
| 1. Egypt | 576 | 8.0 |
| 1. France | 9 | .1 |
| 1. Palestine | 89 | 1.2 |
| 1. Germany | 9 | .1 |
| 1. India | 491 | 6.9 |
| 1. Indonesia | 47 | .7 |
| 1. Iran | 5 | .1 |
| 1. Iraq | 100 | 1.4 |
| 1. Ireland | 4 | .1 |
| 1. Jordan | 80 | 1.1 |
| 1. Kuwait | 576 | 8.0 |
| 1. Lebanon | 67 | .9 |
| 1. Libya | 108 | 1.5 |
| 1. Malaysia | 138 | 1.9 |
| 1. Mauritania | 14 | .2 |
| 1. Morocco | 121 | 1.7 |
| 1. Holland | 3 | .0 |
| 1. Nigeria | 288 | 4.0 |
| 1. Sultanate of Oman | 188 | 2.6 |
| 1. Pakistan | 148 | 2.1 |
| 1. Philippines | 215 | 3.0 |
| 1. Qatar | 14 | .2 |
| 1. Saudi Arabia | 919 | 12.8 |
| 1. South Africa | 101 | 1.4 |
| 1. Sudan | 502 | 7.0 |
| 1. Sweden | 67 | .9 |
| 1. Syria | 400 | 5.6 |
| 1. Turkey | 682 | 9.5 |
| 1. Ukraine | 3 | .0 |
| 1. United Arab Emirates | 444 | 6.2 |
| 1. United Kingdom | 225 | 3.1 |
| 1. United States of America | 50 | .7 |
| 1. Yemen | 208 | 2.9 |
| Total | 7164 | 100.0 |
