# Supplementary material for: A multi-country study on the impact of sex and age on oral features of COVID-19 infection in adolescents and young adults
Source: BMC Oral Health. 2022 Nov 19;22:513. doi: 10.1186/s12903-022-02515-5 (PMC9675238; doi:10.1186/s12903-022-02515-5)
Supplement: Supplementary file 2 — Additional file 2. [file 12903_2022_2515_MOESM2_ESM.pdf]

# A multi-country study on the impact of sex and age on oral features of COVID-19 infection in adolescents and young adults

You are invited to participate in this questionnaire, which aims to assess oral lesions' association with COVID-19 infection in adolescents and young adults as well as to identify if sex and age will modify these associations. Participation in thissurvey is not mandatory and you may stop at any time by leaving the survey or closing the browser. The survey takes about 5 minutes. The data will be strictly confidential and will not be accessed except by the research team. For children under 18 years old, guardian consentis required, after which, the questionnaire can be given to the son / daughter to answer the questions themselves. Your participation is highly appreciated. You may further re-send this linkto others to help in spreading the research and improving its outcome.

### Gender

18-23

15-17

11-14

### Consent form

I confirm that I have read and understood the information section. I know that my participation is voluntary and that I am free to end my participation at any time by closing the survey or web browser. I agree to participate in this survey.

Yes

No

### Parent's consent form

I am the parent of the son / daughter: I confirm that I have read and understood the information for the participants in the current study. I know that my son / daughter's participation is voluntary and that he is free to end his participation at any time by closing thesurvey or the web browser without giving any reason. Do you agree that your son / daughter participate in this questionnaire?

Agree

Disagree

## Section 1: Sociodemographic profile

**Gender**

Male

Female

**Your level of education:**

Primary

Intermediate

High school

University degree

**Do you have any medical condition?**

Yes

No

**Maternal education**

Illiterate

Primary\Intermediate

High school

University degree and higher

**Paternal education**

Illiterate

Primary\Intermediate

High school

University degree and higher

**Number of household members** **□1 □2 □ 3 □ 4 □ 5 □ 6 □ 7 □ 8 □ 9 □ ≥10**

**Number of bedrooms** **□1 □2 □ 3 □ 4 □ 5 □ 6 □ 7 □ 8 □ 9 □ ≥10**

## Section II. COVID-19 information

**Have you been infected with COVID-19 ?**

Yes

No

**Have you been suspected to be infected with COVID-19 ?**

Yes

No

**Have anyone of your family members or friend been infected with COVID-19**

Yes

No

## Section III. Oral lesion information

**Do you have any of the following conditions?**

☐None

☐Teeth Discoloration

☐Gum inflammation

☐White lesion

☐Hairy tongue

☐Taste alteration

☐Vomiting /Nausea

☐Wound burns

☐Mouth dryness

☐Mouth airway irritation

☐Cancer
